# What Is the Current Status of Global Health Training for US Postgraduate Trainees in Anesthesiology? What Are Possible Visions for the Future?

**DOI:** 10.1007/s40140-023-00552-6

**Published:** 2023-03-24

**Authors:** Reema I. Sanghvi, Tosha Songolo

**Affiliations:** 1 Division of Global Health, Department of Anesthesiology, University of California San Diego, 200 W. Arbor, San Diego, CA 92103 USA; 2 Global Health and Policy in Anesthesia, San Diego, CA USA

**Keywords:** Global health in anesthesiology, Postgraduate training in global health in anesthesia, Ethics in global health, Global health curriculum, Cross disciplinary, Departmental, and institutional collaboration, ACGME compliant international resident rotations

## Abstract

**Purpose of Review:**

There is a great deal of interest in global health at all levels of educational attainment. Many residency programs wish to offer a global health opportunity (GHO) but lack the resources to create one de novo. This review will look at the prevalence of global health education in residency and consider the fellowships available. It will summarize the existing recommendations about a curriculum in global health and how best to prepare trainees for a GHO.

**Recent Findings:**

While approximately 45% of residency programs make GHOs available to their residents, there is a lack of standardization of curriculum. Logistical and ethical challenges, funding, and the lack of international partners are all barriers to offering a GHO. Residents and fellows can benefit from a GHO as it helps achieve their ACGME core competencies, among other benefits.

**Summary:**

We make the recommendation for more robust training and education with the investment of fewer resources by aligning with existing global health participation opportunities. We also recommend the use of the Consortium of Universities for Global Health (CUGH) which provided curriculum for creating a context in global health for trainees regardless of discipline.

## Introduction

There is an increasing interest in global health education at all levels of educational attainment, and anesthesiology residents are no exception. Medical schools such as ours have global health tracks for interested medical students, and many offer the opportunity to have international field experiences [1]. These experiences have been referred to in the literature in a variety of ways: international rotations, global health outreach, global health opportunities, “in country” experiences, or international resident electives. We will use the term GHOs (global health opportunities) to refer to any of the above.

The reasons for this interest in global health are many. Current residents are part of the millennial generation, born between 1981 and 1996. Millennials have long been recognized to desire meaningful work and to value experiences over things [2]. They also insist on a work life balance that they did not see their baby boomer parents enjoying. This generation is very interested in global health work. They are driven by a desire to see health equity globally (anecdotal evidence from interviewing applicants as the director of a fellowship program in global health).

The definition of global health bears a little discussion. Global health is “a field dedicated to addressing medical problems that transcend national boundaries” [1]. It is also about addressing inequities in health care, whether they occur internationally or locally [3]. During the COVID-19 pandemic, when international travel for global health was neither feasible nor advisable, it became reasonable to remember that health inequities that beg to be addressed occur in our communities just as much as they occur internationally [4]. Global means truly that, not just outside our nation.

Given this burgeoning interest in global health and its study at the postgraduate level in US medical education, we undertook to review the available literature and present what is new. We systematically evaluate the true prevalence of global health electives in anesthesiology residency programs, consider the benefits of having such electives, and enumerate the barriers to offering such GHOs including the ethical concerns inherent to such work. Recommendations for trainee preparation comprising predeparture preparation and post-trip debriefing will be made. We will explore the many fellowship opportunities that exist, and in conclusion, we also offer our thoughts on what the path forward should look like in view of the current trends in global health, including that of decolonizing global health and encouraging collaborations across institutions and disciplines. We offer suggestions so as to minimize the burden on partners in low- and middle-income countries (LMICs) while maximizing the benefit of international exchange for both partners, the US postgraduate programs and their trainees, as well as our colleagues at host institutions in LMICs.

## Search Strategy

A PubMed search using the MeSH terms global health AND anesthesiology AND education revealed 11 papers. Two papers specific to surgery, not anesthesia, were discarded. Papers older than 5 years ago were only considered if determined to be of great significance. References from the reviewed papers were utilized to make necessary points. Additional papers from other disciplines were referenced as necessary.

## Estimating the True Prevalence of Global Health (GH) Electives

The findings from the most recent survey-based research paper about the importance of global health in postgraduate training in anesthesiology [5] are reviewed in some detail here since many of the other survey-based research articles make very similar points. The survey was sent to 158 postgraduate training programs with instructions to forward it to current trainees and junior faculty less than 5 years out from training. As with the other survey-based instruments, the number of responses was suboptimal. It is impossible to know how many people were eligible to receive the survey. Only 101 responses were received. One would have to assume that this represents a subset of the surveyed who were interested enough in global health to open an email with “global health survey” in the subject line. This becomes apparent when we consider the results. Seventy-six percent of the respondents had prior global health experience (23% research, 75% clinical, 66% medical missions, 34% medical education coursework, and 32% non-medical experiences). This experience was obtained by the respondents in college 40% of the time, in medical school 64% of the time, in residency 38% of the time, and in fellowship 12% of the time. Seventy-six percent of the respondents thought their global health experience was important for their personal and academic development. Thirty percent of the respondents said that the availability of a GHO influenced their decision of medical school, 43% their choice of residency program, and 29% their choice of fellowship, and 54% had ranked a program higher because it afforded global health opportunities. Eighty-eight percent were of the opinion that global health electives should be incorporated into anesthesia residencies. Eighty-three percent saw themselves incorporating global health into their post residency work, claiming that they had increased perspective, an opportunity for growth, and increased depth to their training by virtue of having had a GHO.

A survey-based study of the attitudes of program directors [1] had 44% of program directors respond to their email survey. Of those who responded, 61% had a global health elective. Of those who did not, 45% wanted to offer one. Of the global health electives that existed, 77% had articulated educational goals but had a heterogeneity of curricula. Based on findings such as these, we will make the recommendation for more collaboration among institutions so as to standardize curricula and educational goals and not waste resources reinventing the wheel. CUGH offers a free online curriculum that is not discipline specific, complete with educational goals and resources.

An older article utilized a survey-based tool disseminated via residency programs to newly matched residents [6]. Seventy-four percent of programs distributed the survey. Fifty-three percent of polled participants returned completed surveys. While only 15% of the respondents listed the existence of global health opportunities as one of the top 7 (out of 10) reasons for choosing a residency program, 73% of the respondents expressed an interest in doing GHOs and 50% were interested in incorporating global health into their future careers. Those applicants with previous global health experience or those interested in GHOs were more likely to choose a residency program with global health opportunities present during residency. Thirty-five percent were willing to use some of their vacation time to participate in a GHO, and 41% were willing to partially or fully finance it.

Recognizing the potential bias of survey-based studies with such low and varied rates of response, Prin et al. conducted both a survey-based assessment and a web search assessment of whether anesthesiology programs offered GHOs. In their survey sent to 122 residency programs, their response rate was 45.9% with 39 (69.6%) of these programs offering international electives. Interestingly, not all programs with electives listed them on their websites, and when program websites were scoured for the existence of such GHOs, 33.6% of all program websites described such offerings [7].

An older article using survey-based methodology demonstrated that 91% of anesthesia residents surveyed indicated an interest in GHOs, of whom fewer than half (44%) had participated in a GHO. Seventy-nine percent reported that GHO participation affected their current practice or education and 33% commented they were now less wasteful with supplies and resources. Permission from work or obtaining work coverage was the primary barrier for both those with and without previous GHO participation. Of all respondents, 78% agreed that the availability of a GHO residency track would influence their ranking of that program for training, and 71% would pursue a GHO fellowship if available [8]. That having been said, there are now many fellowships available (Table 1), and anecdotally, they often have unfulfilled spots.

**Table 1:** Currently available GH fellowships in anesthesiology

University	Duration	Maximum duration of field work	Funding mechanism	Opportunity to earn advanced degree	Existed since	Website
Boston Children’s	1 year	6 months	Working as faculty in OR	No	2013	https://www.childrenshospital.org/departments/anesthesia
Columbia University	1 year	No information available	Working as faculty in OR	No information available	Proposed for 2018–2019	https://www.anesthesiology.cuimc.columbia.edu/global-anesthesiology-fellowship
Dalhousie	1 year	No information available	No information available	Yes	2016	https://medicine.dal.ca/departments/department-sites/anesthesia/education/global-health.html
Duke University	2 years	12 months	Working as faculty in OR	MS Global Health	2011	https://hyc.globalhealth.duke.edu/programs/global-health-pathway/subspecialties/anesthesiology/
Harvard University	2 years	Research only, no clinical	No information available	MPH	No information available	https://www.pgssc.org/anesthesia-research-fellow
Stanford University	1 year	Up to 12 weeks	Working as faculty in OR	No information available	2012	https://med.stanford.edu/globalanesthesia/education0.html#global_anesthesiaforfellows
UCSD	1 year	6 months	Working as faculty in OR for 11 weeks	Masters in Global Health, to be arranged separately, extra year	2018	https://medschool.ucsd.edu/som/anesthesia/education/fellowships/Pages/Global-Health-and-Policy-Fellowship.aspx
UCSF	1 year/2 years if Masters	Up to 6 months	Working as faculty in OR for 6 months	Masters in Global Health, optional, extra year	2015	https://anesthesia.ucsf.edu/global-health-equity-fellowship
UCSF, HEAL Initiative	2 years	Up to 6 months	HEAL initiative	Masters in Global Health	2015	https://anesthesia.ucsf.edu/divisions/division-global-health-equity#fellowships
University of Ottawa	1 year	No information available	No information available	No information available	No information available	https://med.uottawa.ca/anesthesiology/education-and-training/fellowships-anesthesiology/global-health
University of Washington	1–2 years	Up to 7 months	Working as faculty in OR for 5 months	MPH Global Health if 2 years	2012	https://depts.washington.edu/anesth/education/fellows/globalhealth.shtml
Vanderbilt University	1–2 years	Up to 6 months	Working as faculty in OR for 4 months	MPH if 2 years	2014	https://www.vumc.org/anesthesiology/global-anesthesiology-fellowship
Weill Cornell Medical College	1 year	3 months	Working as faculty in OR	Unclear	2014	https://anesthesiology.weill.cornell.edu/education/global-health-initiative/fellowship-program

## Barriers to Offering GHO

Some of the factors that limit the ability of a residency program to support the offering of a GHO are as follows: a lack of international partners, a lack of a long-term relationship with said international partners, a lack of finances for support of resident and faculty travel, a lack of finances to grant faculty the time away from their clinical obligations to pursue such involvement, and the logistical difficulties in relieving the residents of their duties to the home institution [6, 8]. The host institutions incur great burdens in having to provide adequate supervision of US trainees [9, 10]. The most successful programs are well funded with grants and governmental support, have the support of the receiving country’s Ministry of Health, and are long-term partnerships [11]. The few international sites that do entertain such engagement are often overwhelmed with having residents from many different institutions come year round [12]. Visitor fatigue sets in, and the staff have confided to me as our personal relationships developed (and they had a drink or two in them) that they wish sometimes that it were just them working there and that they would not have to always share their space with strangers and explain everything repeatedly to new people. Repeat visits are the most welcome [13].

Setting up appropriate mutual expectations of engagement and supervision for a GHO is critical and best done in the context of a long-term relationship. When a Norwegian university offered to help create capacity in Ethiopia by starting a residency program, it took 2 years of discussion before they even began sending Norwegian faculty [14]. Short-term engagements result in difficulty finding adequate supervision in the host country. The ideal way to ensure that trainees have adequate supervision on their GHOs is to have them be accompanied by their own faculty who can serve to provide context and be role models.

Another barrier to offering GHOs is that fairness and the injunction to decolonize global health dictate that there be a bilateral exchange and that trainees not flow solely from HICs (high-income countries) to LMICs (low- and middle-income countries). While that is advisable, it often falls into the category of aspirational. Some US institutions have in fact succeeded in hosting international trainees in a bilateral exchange for observerships [15, 16]. One imagines that they come with a visitor visa, jump through the sorts of hoops a volunteer at any hospital has to jump through, but that they are not able to treat patients since it is not possible to obtain a license to practice medicine in the US without having trained here. On the other hand, the partnership of the American Society of Anesthesiologists Global Humanitarian Outreach (ASA-GHO) committee with the Canadian Anesthesiologists Society International Education Foundation (CASIEF) to support a residency program in Guyana has benefitted from the ability of our Canadian colleagues to arrange for the Guyanese residents to come do clinical rotations in Hamilton, Ontario, for 4 months. Anecdotally, all the Guyanese residents that have done such rotations are clear that the experience was worthwhile even though it meant leaving their families and being cold and lonely.

Finally, given the lack of financial resources that many programs suffer from to facilitate such GHOs, US trainees and their faculty who choose to partake in such opportunities sometimes have to spend their own money and vacation time [6, 17].

## Benefits of GHOs

There is much written, both in the anesthesiology literature and in that of medical education in other disciplines as well as on the utility of GHOs in helping trainees meet the 6 ACGME core competencies [3, 18–21]. These competencies are as follows: patient care, medical knowledge, professionalism, interpersonal and communication skills, practice-based learning, and improvement and system-based practice. It does not take too much imagination to see how a GHO might enhance one’s acquisition of these skills. Unlike international trainees who travel to the US on the rare bilateral exchanges, US trainees who go overseas participate in patient care and bedside teaching in the operating rooms of the host countries. Their medical knowledge is enhanced by seeing disease processes that they either have no opportunity to see at their home institutions or by seeing patients present much later in the process of having a familiar disease than at their home institutions. Their skills in professionalism are enhanced by having to learn to function in a completely different cultural context with peers and patients [22]. This necessitates the improvement of their interpersonal and communications skills [23]. Practice-based learning is the goal of the GHO, and improvements in system-based practice flow from necessity in this setting of working in another institution, another country, in a system that is resource challenged [18].

It has also been suggested that one of the benefits of GHOs for both practicing anesthesiologists as well as residents is to remind us of why we chose medicine in the first place, to help people in their hour of need [19, 24]. This then serves as an effective antidote to burnout, which is increasingly a problem among US and international anesthesiologists [25]. The intensive nature of the emotional involvement in GHOs [26, 27] is precisely the opposite of burnout, in which one is emotionally numbed by the circumstances of one’s profession and work.

There are also benefits that accrue to residency programs that offer GHOs and to the institutions that offer global health fellowships. Students who are interested in global health and have some experience in it rank a residency higher if it offers them continued global health opportunities [8].

## Ethical Considerations

Firstly, we must consider the burden that is imposed on the host institution when we send our trainees, especially if they are not with accompanying faculty. The staff at the host institution have to serve as translators if our trainees do not speak the local language and often even if they do speak the local language. When one works in Peru and speaks Spanish, one can talk to most of the staff, but not necessarily the indigenous patient population who speak only Quechua, or other tribal languages that are seldom spoken outside the region. The same is true in most host countries where the indigenous patients will often not speak the national language.

Host institutions have to provide lodging, transportation, and sometimes meals, all of which imposes a financial burden upon them. Established international partner host institutions will have enough experience to negotiate this as part of the initial conversations, but host institutions with little experience may be reluctant to ask for help with bearing these burdens.

We must be aware of the power imbalance that exists in every aspect of these relationships, in the clinical arena, in teaching, and in research. Clinically, the hosts have to bear the burden of the visitors’ emotional response to the inevitable bad outcomes that they will encounter and will have the responsibility of consoling the visitors while dealing with their own grief and frustration at those inevitable bad outcomes [13]. In research, it is imperative that the research agenda be set by the host institution. When trainees in Uganda were surveyed about the visiting faculty and trainees, they felt that only 15% of the research that was being done was on things that were a priority for Uganda [12]. Twenty-eight percent of them had been involved in these research projects, yet none had been listed as co-author. In the same paper, as many as 40% of the Ugandan trainees felt that international visitors had a neutral or negative impact on patient care. The conversation on decolonizing global health, which involves examining these long-standing power dynamics, is ongoing and has been much overdue [28].

Anecdotally, US trainees have been known to overstep their training [29, 30]. When host communities in India and La Paz, Bolivia, were surveyed, many of them wished the visiting US trainees’ attitudes and behavior were better [29]. They particularly wanted them to not make promises they would not or could not keep. While many papers [29, 31] include injunctions on trainees overstepping their training, there is not much in the literature of examples of how they might do so. Being put in situations wherein it is possible to overstep their training is less likely to happen when trainees are sent overseas to work and learn with faculty supervision from their home institution. While it is clearly unethical for trainees to overstep their training in LMICs, it must be recognized that the desire to help, which is the motivation for participation in a GHO in the first place, is the root cause of this potential pitfall.

## Trainee Preparation

Appropriate preparation for the trainee is key, as is a thorough debriefing after the experience. Ideally, GHOs should occur in the context of a global health curriculum for trainees. That curriculum should include a larger understanding of global health and policy, of the history of global health, of the evolution of the problem as being one of infectious diseases to being one of interactional diseases (otherwise referred to as non-communicable diseases, [32], of the role of surgery as a cost-effective intervention to prevent death and disability, and of the fact that the lack of access to surgery causes more death than do malaria, tuberculosis, and HIV all put together [33]. There should be an understanding of global health equity [3, 18] and of common themes in global health such as task shifting, brain drain, maternal health, under 5 mortality, and decolonization. An article from the pediatric literature does a wonderful job of listing all of the areas in which preparation is necessary, starting with logistics and safety. Safety comprises personal physical and mental health, travel insurance, evacuation insurance, and registration with the state department. Logistics such as visa and customs, malpractice insurance, and medical licensure “in country” must be attended to [34].

The next category is that of medical skills and knowledge. First, the trainee must be prepared for the types of diseases that are endemic to the area to which they are traveling, including being aware of the prevalence of HIV and hepatitis. It may be advisable to carry post-exposure doses of antiretroviral agents given the risk of needle stick injuries when doing clinical work. He/she should also have a knowledge of the health systems that exist in the host country. Procedural and practice skills that are relevant in the host country should be practiced, by simulation if not in actuality.

Attitudes and behaviors need to be examined. The trainees’ personal motivation for engaging in the GHO should be discussed, and the appropriate attitude of humility should be emphasized. The idea of culture shock and reverse culture shock on return to the USA should be discussed and anticipated. Local resources, clinical and human, should be considered, and it is imperative to have had a discussion with the hosts about specific expectations. Trainees should have an understanding of local culture and laws, history, politics, economics, the prevalent religion and related practices, local health beliefs which may not be biologically based, and, of course, the local language. Provision should be made before departure to arrange for interpretation services as necessary for visiting US trainees, and it should be understood that if this is not done, the local doctors, who are already busy, will have to take on the additional burden of being interpreters and tour guides.

Ethical concerns regarding donations, research, patient privacy, trainee supervision, and scope of practice all have to be anticipated and clearly communicated. Communication should occur predeparture, on site and post return with the trainee so as to be certain that this learning is taking place in an emotionally safe manner wherein the trainee feels supported in learning to cope with this unfamiliar environment. Finally, partnerships have to be well defined with appropriate selection of trainees, expectations clearly outlined in a bilaterally signed memorandum of understanding, and ongoing evaluation of whether these mutual expectations are being met must occur.

## Fellowship Training

In 2015, McGoldrick, who had just completed a fellowship herself at the time, published a paper that included a table of all the GH fellowships that existed at the time [35]. We present Table 1 that lists currently available GH fellowships in anesthesiology.

## Future Directions

Having considered the lack of standardization of global health curricula for postgraduate education in anesthesiology and having considered the enormous amount of resources that it takes for a US residency program to create a long-term international partnership, we would like to offer suggestions on collaborations and partnerships that allow interested residency programs to share resources with the ones that are already offering global health tracks in residency and fellowships.

First are the interdisciplinary collaborations within a given institution. At the University of California at San Diego (UCSD), we are fortunate to have a multidisciplinary initiative started by an obstetrician that includes residents and fellows from the departments of emergency medicine, Ob/Gyne, internal medicine (infectious diseases in particular), surgery, and anesthesiology. Together, the consortium presents didactic material for the residents and fellows in different disciplines four times a year, comprising two simulator sessions and two journal clubs. Weill Cornell Medical School made a concerted effort to integrate global health at every level of medical education and across many disciplines and should inspire more medical schools and universities to make that kind of well-resourced and coordinated effort to promote global health [36]. These are examples of ways that different institutions have relied on intrainstitutional partnerships and collaborations to address a long-standing problem in global health, that of silos. Often global health practitioners are unaware of the work of other global health practitioners within the same institution.

Interinstitutional partnerships and collaborations are emerging and welcome phenomena. The Stanford Anesthesia Division of Global Health Equity offers a global health track to its residents. There is a monthly lecture, and by inviting lecturers from the University of Colorado, UCSD, and the Virginia Mason Medical Center to contribute to the lecture series and making it available to all the anesthesiology residents in California, they have created valuable interinstitutional collaborations. Vanderbilt University has also made its quarterly journal club available to a wide swath of anesthesiology residencies with an interest in global health. Finally, the University of Wisconsin has put together a Global Academic Anesthesia Consortium (GAAC) that brings together 10 or 12 academic institutions and supports their engagement with the residency training program in Zambia.

Finally, there are the long-standing institutions that make their offerings available to academic programs as well as private practitioners. These include the ASA-GHO committee (which began as the ASA-OTP (overseas training program) and will soon be known as the ASA GH (global health) committee). This committee offers scholarships to PGY 4 residents to spend a month at an affiliated international site with their expenses being covered by the ASA [37]. They also support the residency programs in Guyana and Rwanda and collaborate with CASIEF to send volunteers year round to provide in person bedside teaching and didactics as needed to both countries. The ASA GH committee also promotes bilateral exchange by offering scholarships to global scholars from LMICs to attend the ASA annual meeting and be observers at the local universities where the annual meeting is held. The Society for Education in Anesthesia partners with health volunteers overseas (SEA-HVO) to offer month long volunteer opportunities to anesthesiologists, including residents, and many residency programs utilize these avenues to provide their residents with a GHO [19]. Collaboration is the future, and the current Chair of the ASA GH committee makes a passionate and eloquent argument for the importance of these partnerships [27].

The particulars of curriculum can be difficult to come up with for the residency program that does not have the faculty resources to devote to global health, and the CUGH (Consortium of Universities for Global Health) has some help to offer. At https://www.cugh.org/online-tools/competencies-toolkit/, they make available a toolkit that includes global health competencies, teaching strategies to achieve them, and resources to support learning those competencies. The curriculum is comprehensive and covers the global burden of disease, the globalization of health and health care, the social and environmental determinants of health, capacity strengthening, collaboration, partnering and communication, ethics, professional practice, health equity and social justice, program management, sociocultural and political awareness, and strategic analysis. It is not discipline specific and helps with creating the context of global health that is essential for a good GHO. The University of Wisconsin has also created a comprehensive curriculum for a global health rotation that utilizes the structure of the six core competencies of the ACGME that is very useful.

## Conclusion

Interest in postgraduate training in global health among anesthesiologists is robust and growing. There is an increasing awareness caused by the recent pandemic that global health issues and inequities affect us all and that local is global and vice versa. Volunteerism is an important strategy to mitigate burnout, which is a modern plague among health care workers. GHOs are to be encouraged as long as done with a thorough understanding of the many issues of power imbalances between rich country training programs and LMIC institutions that need our support. The way forward is collaborative and paved with partnerships, interinstitutional and interdisciplinary. Decolonizing global health is a worthwhile endeavor that requires constant reflection, by individuals and their institutions as they create partnerships needed to address the larger issue of inequities globally, in health and otherwise. As more training programs in anesthesiology seek to offer their residents an education in global health, we urge them to look to the CUGH resources which apply to all disciplines. Standardization of the curricula will undoubtedly follow as the discipline of global health matures and as GHOs are appropriately contextualized within an understanding of the global burden of disease and global health inequities.
